# Nanowires-Assembled TiO_2_ Nanorods Anchored on Multilayer Graphene for High-Performance Anodes of Lithium-Ion Batteries

**DOI:** 10.3390/nano12203697

**Published:** 2022-10-21

**Authors:** Junming Xu, Dongfang Chen, Jianfeng Wu, Jun Wu, Jijun Zhou, Tao Zhou, Xinchang Wang, Jipeng Cheng

**Affiliations:** 1 College of Electronic Information, Hangzhou Dianzi University, Hangzhou 310018, China; 2 College of Information Science & Technology, Zhejiang Shuren University, Hangzhou 310015, China; 3 School of Physics and Microelectronics, Zhengzhou University, Zhengzhou 450052, China; 4 School of Materials Science & Engineering, Zhejiang University, Hangzhou 310027, China

**Keywords:** TiO_2_ nanowires, TiO_2_ nanorods, multilayer graphene, composites, LIB anodes

## Abstract

Multilayer graphene (MLG) prepared via ultrasonic exfoliation has many advantages such as its low-cost and defect-free nature, high electronic conductivity, and large specific surface area, which make it an apt conductive substrate for TiO_2_ composites. To synthesize graphene/TiO_2_ hybrids, traditional methods that greatly depend on the chemical bond of oxygen-containing functional groups on graphene with titanium cations are not applicable due to the absence of these functional groups on MLG. In this work, a facile chemical method is developed to directly deposit TiO_2_ on the MLG surface without the introduction of chemically active groups. With this method, four types of TiO_2_ materials, that is pure anatase TiO_2_ nanoparticles, a mixture of anatase TiO_2_ nanoparticles and rutile TiO_2_ nanoflowers, pure rutile TiO_2_ nanoflowers, and pure rutile TiO_2_ nanorods, are homogeneously anchored on the MLG surface by controlling the amount of HCl in the reactant. Interestingly, the rutile TiO_2_ nanorods in the TiO_2_/MLG composite are assembled by many TiO_2_ nanowires with an ultra-small diameter and ultra-long length, which provides a better synergetic effect for high performances as LIB anodes than other composites. A specific capacity of 631.4 mAh g^−1^ after 100 cycles at a current density of 100 mA g^−1^ is delivered, indicating it to be a valuable LIB anode material with low cost and high electrochemical performances.

## 1. Introduction

As Lithium-ion batteries (LIBs) have become more and more popularly used for electric vehicles, the enhancement of the energy density of LIBs is desired for longer distances on one charge [1,2]. Anode materials with high specific capacities have been widely researched [3,4] in order to replace commercial graphite, which has a specific capacity of 372 mAh g^−1^. Another shortage of graphite anode is the lithium plating that easily occurs due to the low operation potential, which will cause a serious safety issue [5,6]. Recently, anode materials based on different lithiation mechanisms including insertion, alloying, and conversion were researched [7,8,9,10,11]. As a kind of insertion-type anode material, titanium dioxide (TiO_2_) has many particular advantages due to its high theoretical specific capacity (336 mAh g^−1^), high reversible cyclic stability, and relatively high operational potential. Meanwhile, TiO_2_ has several crystalline phases such as rutile, anatase, and brookite, which can thus provide different electrochemical performances [12,13,14,15,16,17]. However, the poor intrinsic electrical conductivity and weak Li^+^ diffusion capability limit the application of TiO_2_ in LIBs. Reducing the size of TiO_2_ and combining it with highly conductive carbon materials are two efficient ways to improve electrochemical performances [18,19,20,21,22,23,24,25,26].

Graphene is a well-defined 2D material for building TiO_2_ composites due to its large specific surface areas, high electronic conductivity, and superior mechanical performances [27]. Some typical architectures, such as TiO_2_ anchored on graphene [20,21,22], TiO_2_ wrapped by graphene [28], and TiO_2_-modified graphene aerogel [29], were prepared and studied. Among them, TiO_2_ anchored on graphene was intensely studied because of the intimate contact between TiO_2_ and graphene, nano-structured TiO_2_, and 3D conductive graphene networks, thus obtaining a high electrochemical performance of composites. For example, D.Y. Zhao et al. [30] reported the synthesis of TiO_2_ nanoparticles on graphene by a sol-gel method. The resultant TiO_2_ nanocrystals were sized ∼5 nm and ultra-dispersed on graphene, which resulted in a high specific surface area of ∼229 m^2^ g^−1^. The composite exhibited an excellent rate of capability and cycle performance as an anode material for LIBs. Lu Liu et al. [18] reported mesoporous nanoplates of the TiO_2_/reduced graphene oxide composite. The novel mesoporous nanoplates could provide extra space to accommodate the volume changes, shortening the transport length for Li^+^ and e^−^ and superior interfacial kinetics during lithium insertion-extraction. Zheng Jiao et al. [31] synthesized mesoporous succulent-like TiO_2_/graphene aerogel composites. The mesoporous TiO_2_ submicron flowers offered more active sites for lithium ions. Hence, the succulent-like TiO_2_/GA composite exhibited excellent electrochemical properties. The above reports indicate that different morphologies, structures, and crystalline phases of TiO_2_ show different electronic structures and surface atomic geometries, leading to different movement speeds of Li^+^ and e^−^, and thus determining the rate capability and cycling stability. However, controlling the morphology and crystal phase of TiO_2_ in the same system to obtain an optimal TiO_2_/graphene composite for high electrochemical performances has not been reported yet.

On the other hand, in previous research work, GO was popularly used as a substrate for a TiO_2_-graphene composite due to the rich oxygen-containing function groups, which can help the anchor of the Ti cation [18,19,20,21,22]. However, the oxidation treatment of graphite usually needs strong oxidants, such as KMnO_4_ and H_2_O_2_. The severe oxidation of graphene will damage the carbon bonds, thus significantly decreasing the electronic conductivity. Mechanically exfoliated multilayer graphene (MLG) has the advantages of low cost and defects, high electronic conductivity, and large specific surface area, which makes it an ideal substrate for TiO_2_-based hybrids. However, it is still a great challenge to homogeneously anchor TiO_2_ on multilayer graphene uniformly due to the absence of active sites to chemically bond Ti cations. In this work, a facile chemical method is developed to deposit TiO_2_ on MLG surfaces at a low temperature. Moreover, four types of TiO_2_ with different phases and morphologies are controllably synthesized on MLG surfaces. Among them, TiO_2_ nanorods/MLG exhibits the best electrochemical performances as LIB anodes due to their special hierarchical nanostructure.

## 2. Experimental Section

### 2.1. Preparation of Samples

Titanium trichloride (TiCl_3_), hydrochloride (HCl), nitric acid (HNO_3_), *N*,*N*-dimethylformamide (DMF), ethanol and *N*-methylpyrrolidone (NMP) were purchased from Sinopharm Chemical Reagent Co., Ltd. (Shanghai, China) and used as received. Expanded graphite was purchased from Tianyuanda graphite Co., Ltd. (Qingdao, China).

A mechanically exfoliated MLG suspension was prepared by ultrasonicating 20 mg of expanded graphite in a mixed solvent of 4 mL of DMF and 1 mL of distilled water (H_2_O) for 4 h using an ultrasonic cleaner VGT-1990QTD (Suzhou Jiangdong Precision Instrument CO., Ltd., Suzhou, China) with ultrasonic power of 200 W and a frequency of 40 kHz. Subsequently, 360 μL of TiCl_3_ and different amounts of HCl (3.0 mL, 3.5 mL, 4.5 mL, and 6 mL) were added to the suspension at room temperature. It was then magnetically stirred at 90 °C with a rotation speed of 500 rpm for 4 h in a constant-temperature magnetic stirrer HCJ-4A (Jintan Zhiqian Xuri Experimental Instrument Factory, Changzhou, China). Then, 120 μL of HNO_3_ solution (10% vol) was poured into the suspension. After a 2 h reaction, the vial was taken out and cooled in the air. The product was washed several times with water and ethanol by centrifugation with a rotation speed of 6000 rpm. The powder was collected after drying at 70 °C for 24 h. TiO_2_-NP/MLG, TiO_2_-NPNF/MLG, TiO_2_-NF/MLG, and TiO_2_-NR/MLG were used to name the as-synthesized powder in volumes of 3.0 mL, 3.5 mL, 4.5 mL, and 6 mL of HCl, respectively. A pristine TiO_2_ nanorod (TiO_2_-NR) was synthesized by the same procedures of TiO_2_-NR/MLG but without MLG in the solution. MLG powder was obtained by washing and drying the MLG suspension.

### 2.2. Materials Characterization

X-ray diffraction (XRD) was performed on Shimadzu XRD-6000 (Kyoto, Japan) using Cu Kα radiation at a scan rate of 4°/min to analyze the phase composition of samples. Field emission scanning electron microscope (FESEM) and transmission electron microscope (TEM) observations were carried out on a Hitachi S-4800 (Tokyo, Japan) and a JEOL-2100F (Tokyo, Japan), respectively.

### 2.3. Anode Performance Measurements for LIBs

As-synthesized samples were used to fabricate working electrodes. The sample material, conductive carbon, and poly(vinyldifluoride) (PVDF) (Shanghai Sanaifu New Material Technology Co., Ltd, Shanghai, China) were mixed in NMP as the solvent with a weight ratio of 80:10:10 and stirred at room temperature for 3 h. Then the slurry was pasted onto copper foils and dried in a vacuum oven at 80 °C for 20 h. The working electrodes were then assembled into coin-type lithium-ion batteries with lithium foils as counter/reference electrodes and a solution of 1 M LiPF_6_ in ethylene carbonate (EC)/dimethyl carbonate (DMC) (1/1, *w*/*w*) (Tianjin Jinniu Power Sources Material Co., Ltd.) as the electrolyte. Assembling cells were operated in an argon-filled glove box MIKROUNA, Super (1220/750/900) (Shanghai, China), where both moisture and oxygen levels were less than 1 ppm. A CHI-660E electrochemical workstation (Shanghai, China) was used to test cyclic voltammetry (CV) curves of the cells at a scan rate of 0.1 mV/s and electrochemical impedance spectroscopy (EIS) with a perturbation voltage of 10 mV in a frequency range of 10 kHz to 0.01 Hz. A NEWARE multi-channel battery test system CT-ZWJ-3′S-T (Shenzhen China) was used to test galvanostatic charge/discharge (GCD) processes and cycling performances of cells in a voltage range of 0.01–3.0 V.

## 3. Results and Discussion

**Figure 1:**
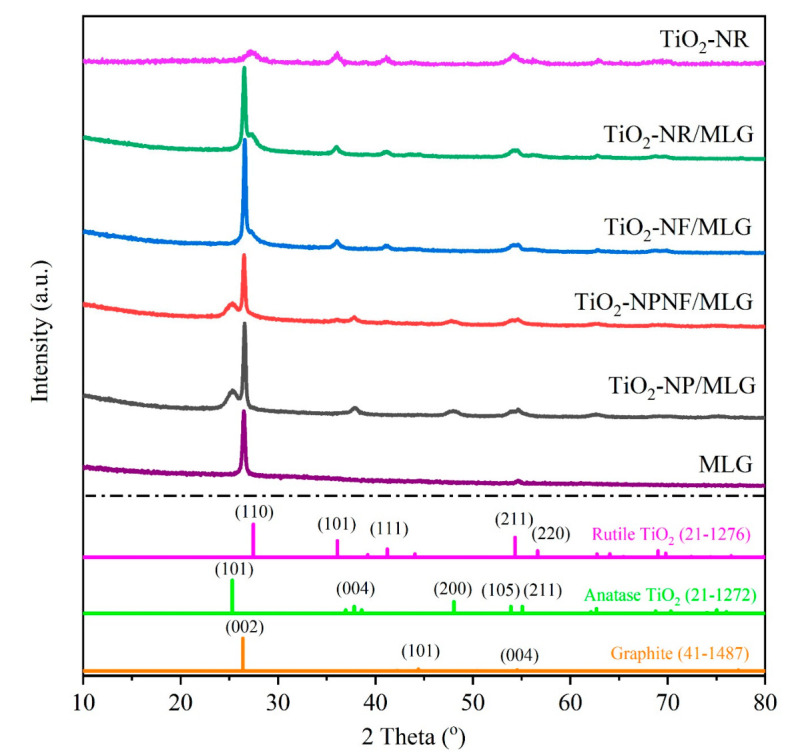
XRD patterns of TiO_2_/MLG composites, MLG, and TiO_2_-NR.

The XRD patterns of MLG, four TiO_2_/MLG composites, and pure TiO_2_-NR are shown in Figure 1. The strong peak at 26.5° belongs to the (002) plane of graphite (No. 41-1487), indicating the graphitic layer structure of MLG. The layer number of MLG was measured to be approximately 10 via AFM measurement [8]. In TiO_2_/MLG composites, all diffraction peaks belong to the TiO_2_ phase. Notably, TiO_2_ gradually transforms from anatase (PDF No. 21-1272) into the rutile phase (PDF No. 21-1276) with an increasing amount of HCl from 3.0 mL to 4.5 mL. The pure anatase phase, a mixture of anatase and rutile phases, and the pure rutile phase are determined in TiO_2_-NP/MLG, TiO_2_-NPNW/MLG, and TiO_2_-NF/MLG composites, respectively. The crystalline TiO_2_ in TiO_2_-NR/MLG is also in the rutile phase, meaning that the rutile phase is maintained for HCl over 4.5 mL. Pure rutile TiO_2_-NR has a similar XRD pattern to the TiO_2_-NR/MLG sample except for the peak at 26.5°, which belongs to MLG, indicating that the addition of MLG does not change the crystalline phase of TiO_2_ and just provides the substrate for TiO_2_ nanostructures.

**Figure 2:**
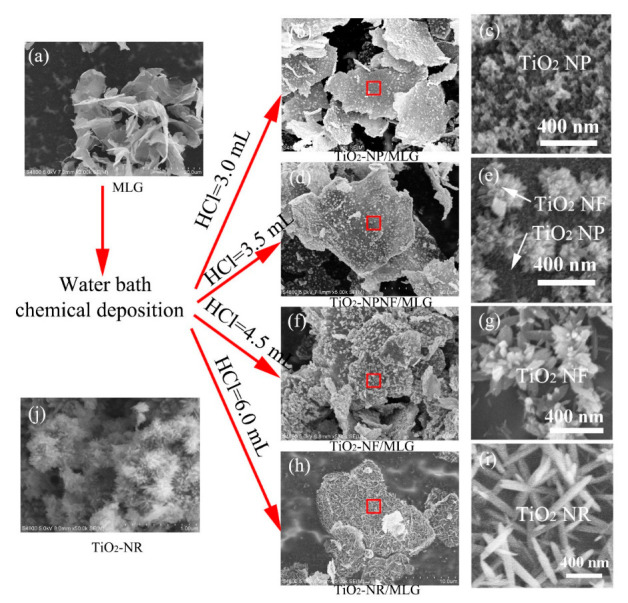
SEM images of MLG (**a**), TiO_2_-NP/MLG (**b**,**c**), TiO_2_-NPNF/MLG (**d**,**e**), TiO_2_-NF/MLG (**f**,**g**), TiO_2_-NR/MLG (**h**,**i**), and TiO_2_-NR (**j**). Small red rectangles in (**b**,**d**,**f**,**h**) mark the places for (**c**,**e**,**g**,**i**), respectively.

The typical SEM images of MLG, four TiO_2_/MLG composites, and TiO_2_-NR are shown in Figure 2. Figure 2a shows the SEM image of MLG. It can be observed that MLG is ultra-thin in thickness and smooth on the surface. A few oxygen-containing groups are present on the surface of MLG, which have been determined by XPS in our previous report [32]. Then, with our developed chemical method, four types of TiO_2_ nanostructures are anchored on MLG tuning by the different amounts of HCl. Both Figure 2b,c show the SEM images of TiO_2_-NP/MLG, in which 3.0 mL of HCl is added. A thin layer of anatase TiO_2_ nanoparticles is homogeneously distributed on the MLG surface. Figure 2d,e are the SEM images of TiO_2_-NPNF/MLG, where 3.5 mL of HCl is used. Thin films consisting of anatase TiO_2_ nanoparticles and rutile TiO_2_ nanoflowers are decorated on the surface of MLG. As shown in Figure 2f,g, when HCl is increased to 4.5 mL, only rutile TiO_2_ nanoflowers are anchored on the surface of MLG, and TiO_2_ nanoparticles disappear when compared with TiO_2_-NPNF/MLG. Figure 2h,i exhibit the SEM images of TiO_2_-NR/MLG, where HCl is further increased to 6 mL. It can be observed that a large number of rutile TiO_2_ nanorods are anchored on the MLG surface. The average diameter of nanorods is approximately 80 nm and the mean length mostly ranges from 400 nm to 550 nm. These TiO_2_ nanorods construct a 2D network on the MLG surface due to their interconnection. Figure 2j displays the SEM images of TiO_2_-NR. Homogenous nanorods with diameters of approximately 15 nm to 20 nm are randomly aggregated together. It indicates that TiO_2_ nanorods cannot grow to a large size as in TiO_2_-NR/MLG due to the absence of substrates. From the above XRD and SEM results, we can find that the phase and morphology of TiO_2_ nanostructures anchored on MLG are greatly influenced by the amount of HCl in the reactant.

**Figure 3:**
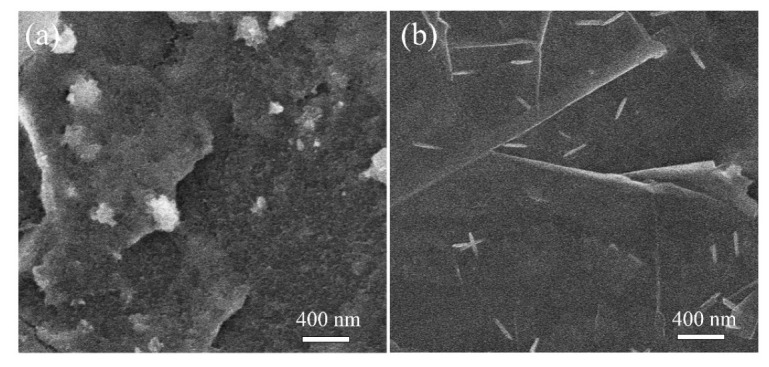
SEM images of TiO_2_-NP/MLG (**a**) and TiO_2_-NR/MLG (**b**) in the stage before the addition of HNO_3_.

In order to understand the growth mechanism of TiO_2_ on MLG, the intermediate products of TiO_2_-NP/MLG and TiO_2_-NR/MLG were both observed with SEM. Figure 3a,b show the SEM images of TiO_2_-NP/MLG and TiO_2_-NR/MLG in the stage before the addition of HNO_3_, respectively. It can be observed that many TiO_2_ nuclei are anchored on the MLG surface in this stage. These TiO_2_ nuclei are derived from the hydrolysis of TiCl_4_, which is from the oxidation of TiCl_3_ by oxygen. So, the crystal phase and morphology of TiO_2_ can be controlled by the amount of HCl, which can suppress the hydrolysis process [33]. When HCl is 3.0 mL, the hydrolysis rate is fast, thus leading to the formation of the metastable anatase phase of TiO_2_ with a high nucleation rate. These TiO_2_ nuclei are ultra-small with low crystallization and large in quantity (in Figure 3a). On the contrary, when HCl is 6.0 mL, the stable rutile phase of TiO_2_ can be obtained due to the slow hydrolysis rate. These TiO_2_ nuclei have a nanorod shape, are better crystallized, and are in a smaller quantity than that using 3.0 mL HCl (Figure 3b). After the addition of HNO_3_, the remaining TiCl_3_ is then oxidized to TiCl_4_ and hydrolyzed to form TiO_2_, resulting in the further nucleation and growth of TiO_2_ to nanorods.

**Figure 4:**
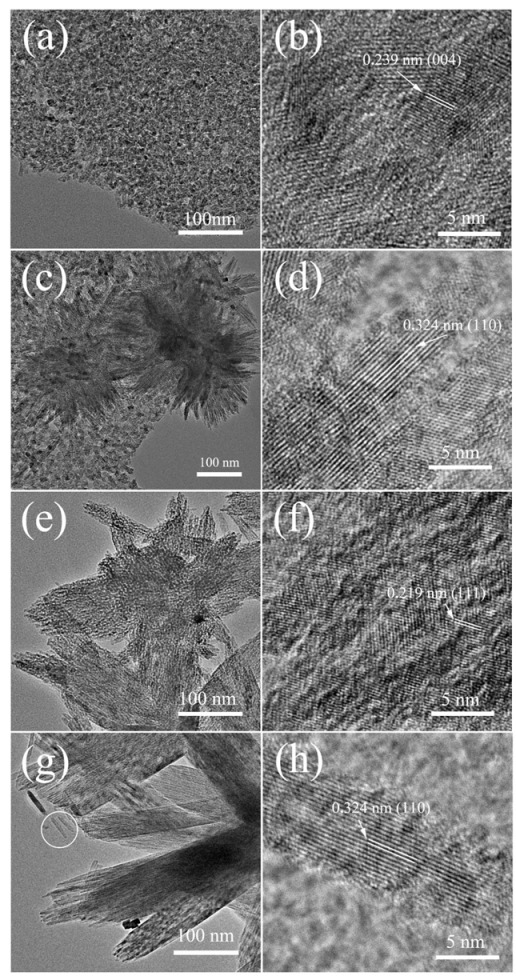
TEM and HRTEM images of TiO_2_-NP/MLG (**a**,**b**), TiO_2_-NPNF/MLG (**c**,**d**), TiO_2_-NF/MLG (**e**,**f**), and TiO_2_-NR/MLG (**g**,**h**).

Four TiO_2_/MLG composites were also observed by TEM in order to further understand the detailed structures. Regarding the TiO_2_-NP/MLG composites in Figure 4a,b, they show that a dense layer of TiO_2_ nanoparticles, homogeneous in size with a diameter of approximately 7 nm, completely coats MLG surfaces. A high-resolution TEM (HRTEM) image (Figure 4b) shows an interlayer distance of 0.239 nm, which corresponds well with the (110) planes of anatase TiO_2_. Figure 4c is a TEM image of TiO_2_-NPNF/MLG. A dense layer consisting of TiO_2_ nanoparticles and nanoflowers is observed on the surface of MLG. These TiO_2_ nanoparticles are approximately 10 nm in size, which is larger than that in TiO_2_-NP/MLG. The nanoflowers are composed of many TiO_2_ nanoneedles, which are scattered from the center region. A typical high-resolution TEM image of the top region of a nanoflower is exhibited in Figure 4d, displaying sufficient crystallization in the center and poor crystallization on the surface. An interlayer distance of 0.324 nm is assigned to the (110) planes of rutile TiO_2_, indicating that the TiO_2_ nanoflowers are rutile in phase. Figure 4e,f show the TEM and HRTEM images of nanoflowers in the TiO_2_-NF/MLG sample, respectively. Many pores can be observed in the nanoflowers, and the interlayer distance of 0.219 nm belongs to rutile TiO_2_ (111) planes. Figure 4g is a TEM image of nanorods in the TiO_2_-NR/MLG sample. It can be clearly observed that the rods are composed of many sub-sized nanowires, which have the same length as nanorods but are much smaller in diameter, measuring approximately 7 nm. These nanowires are packed together with small gaps. An HRTEM image of a separated nanowire marked in a cycle in Figure 4g is shown in Figure 4h. It shows that the nanowire is well crystallized and has an interlayer distance of 0.324 nm belonging to the rutile TiO_2_ (110) plane.

**Figure 5:**
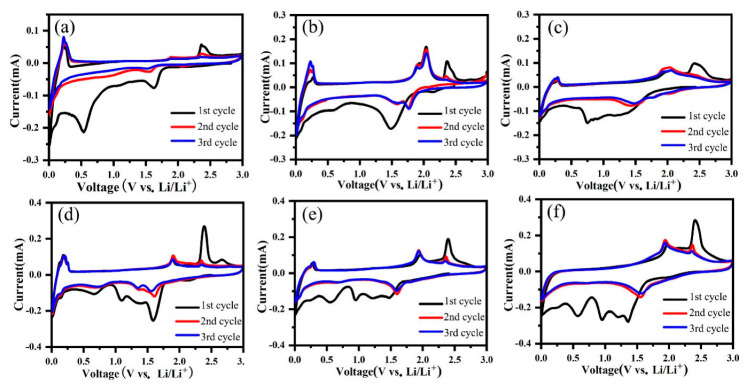
CV curves of MLG (**a**), TiO_2_-NP/MLG (**b**), TiO_2_-NPNF/MLG (**c**), TiO_2_-NF/MLG (**d**), TiO_2_-NR/MLG (**e**), and TiO_2_-NR (**f**).

Electrochemical performances of the assembled semi-cells with these materials as anode electrodes were tested. Figure 5 shows the CV curves of all the samples in the initial three cycles. In the first cycle for the MLG electrode (in Figure 5a), a cathodic peak at 1.62 V and an anodic peak at 2.35 V relate to the irreversible lithium storage due to the defects of MLG, and both are gradually weakened in the following two cycles and almost disappear after three cycles. The cathodic peak at 0.54 V is attributed to the formation of SEI films on the active material in the first cycle. The cathodic and anodic peaks below 0.31 V are attributed to the insertion and extraction of the lithium ion in the graphitic layer of MLG, which is reversible during the following cycles. For all TiO_2_/MLG composites (Figure 5b–d), the broad peaks between 1.75 V and 0.5 V appeared in the first cathodic process, which may be attributed to the insertion of lithium ion into TiO_2_, the interfacial storage of the lithium ion, and the formation of SEI films. The difference in the first cathodic profiles of four TiO_2_/MLG composites may be related to their different phases and morphologies of TiO_2_, which leads to different specific surface areas and electrochemical activity of TiO_2_. The anodic peaks at approximately 2.34 V can be attributed to the irreversible lithium-ion storage due to the defects of TiO_2_ and MLG, which become gradually weaker in the following cycles. From the second cycle, the redox reaction is highly reversible, which shows stable cathodic peaks at 1.59 V–1.77 V and anodic peaks at 1.88 V–2.05 V deriving from the reversible reaction (TiO_2_ + xLi^+^ + xe^−^↔ Li_x_TiO_2_) [30]. The peaks below 0.31 V can be observed in all TiO_2_/MLG composites, representing the contribution of MLG to lithium-ion storage in the composites. Pure TiO_2_-NR has similar CV profiles to TiO_2_-NR/MLG except for the peaks from MLG (Figure 5f).

**Figure 6:**
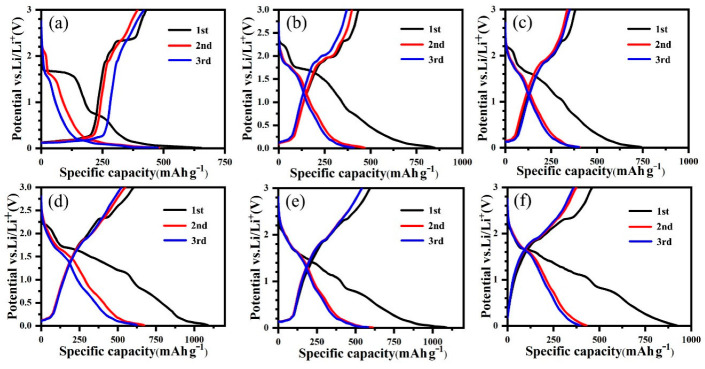
GCD curves of MLG (**a**), TiO_2_-NP/MLG (**b**), TiO_2_-NPNF/MLG (**c**), TiO_2_-NF/MLG (**d**), TiO_2_-NR/MLG (**e**), and TiO_2_-NR (**f**).

GCD curves of all cells in the first three cycles are displayed in Figure 6. The GCD profiles agree well with the CV curves. As shown in Figure 6a, MLG shows an initial discharge capacity of 652 mAh g^−1^ and a charge capacity of 429 mAh g^−1^ in the first cycle. The irreversible capacity can be caused by the defects of MLG as well as the formation of SEI films. In the third cycle, the discharge and charge capacities are 475 mAh g^−1^ and 419 mAh g^−1^, respectively, which mostly come from the insertion and extraction of lithium in the graphitic layer of MLG. A plateau at approximately 1.71 V in the first discharge process of TiO_2_-NP/MLG (Figure 6b) may be attributed to the insertion of the lithium ion into anatase TiO_2_ [30]. Then, TiO_2_ will react with lithium ions when the potential is below 1.0 V, which destroys the crystal structure of anatase TiO_2_, resulting in the disappearance of the plateau in the following cycle. As shown in Figure 6c–e, TiO_2_-NPNF/MLG, TiO_2_-NF/MLG, and TiO_2_-NR/MLG samples do not have obvious plateaus, such as in TiO_2_-NP/MLG in the first discharge curve. This difference may be caused by the crystal structural difference between the anatase and rutile phases of TiO_2_ [34]. In the following second and third cycles, four TiO_2_/MLG samples have similar discharge and charge curves, indicating similar electrochemical processes after activation despite their initial crystal phases and morphologies. The TiO_2_-NR/MLG sample delivers a discharge capacity of 1088 mAh g^−1^ and a charge capacity of 594 mAh g^−1^ in the first cycle, showing a columbic efficiency of 54.6%. The columbic efficiency is increased to 88.7% in the second cycle, showing high reversibility after one work cycle. TiO_2_-NR shows similar discharge and charge curves to TiO_2_-NR/MLG except for a short capacity below 0.3 V due to the absence of MLG. A discharge capacity of 921.9 mAh g^−1^ and a charge capacity of 460.7 mAh g^−1^ are delivered in the first cycle for pure TiO_2_-NR, showing a rather low columbic efficiency of 50.0%.

**Figure 7:**
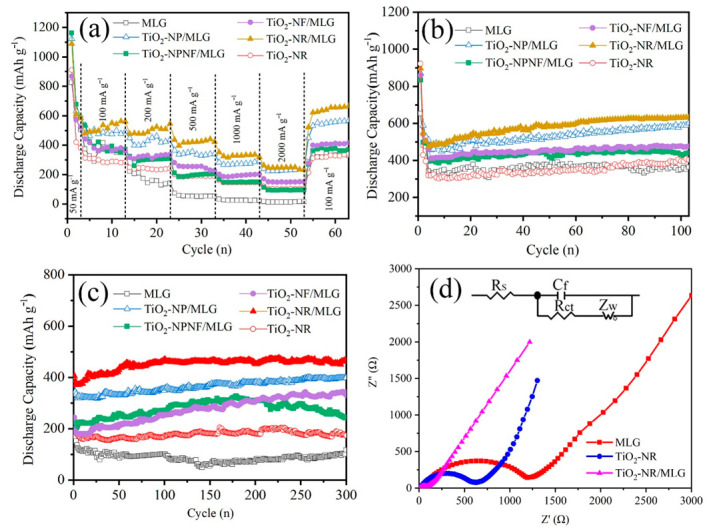
(**a**) Rate performances of all samples at different current densities, (**b**) cycling performances of all samples at 0.1 A g^−1^, (**c**) cycling performances of all samples at 0.5 A g^−1^, (**d**) Nyquist plots of MLG, TiO_2_-NR/MLG, and TiO_2_-NR.

Figure 7a shows the rate performances of the samples under increased current densities from 0.1 A g^−1^ to 2 A g^−1^. It is obviously observed that TiO_2_-NR/MLG has the highest capacities at different current densities. A discharge capacity of 547.5 mAh g^−1^ is delivered for TiO_2_-NR/MLG at 0.1 A g^−1^. Then the capacity continuously decreases with the gradual increase in current density. A discharge capacity of 248.8 mAh g^−1^ is retained at 2 A g^−1^, indicating its high-rate performance. As a comparison, MLG delivers 347.6 mAh g^−1^ at a current density of 0.1 A g^−1^, and rapidly decreases to 51.3 mAh g^−1^ at a current density of 0.5 A g^−1^. TiO_2_-NR exhibits capacities of 282.2 mAh g^−1^ and 102.2 mAh g^−1^ at 0.1 A g^−1^ and 2 A g^−1^, respectively. The capacity of the TiO_2_-NR/MLG composite is much higher than those of MLG and TiO_2_-NR at every current density, indicating a sufficient synergic effect between TiO_2_ nanorods and MLG in the composite.

TiO_2_-NP/MLG, TiO_2_-NPNF/MLG, and TiO_2_-NF/MLG deliver specific capacities of 473.4 mAh g^−1^, 347.6 mAh g^−1^, and 359.2 mAh g^−1^ at a current density of 0.1 A g^−1^, respectively, and 230.3 mAh g^−1^, 90.6 mAh g^−1^, and 147.7 mAh g^−1^ at 2 A g^−1^, respectively. It can be concluded that the specific capacities of TiO_2_-NF/MLG, TiO_2_-NP/MLG, and TiO_2_-NR/MLG increased in order. This phenomenon may be related to the number of accessible active sites in the electrodes. For TiO_2_-NP/MLG, ultra-small TiO_2_ nanoparticles on MLG enable fast electron transfer. However, the small size and close arrangement of TiO_2_ nanoparticles on MLG leave small gaps between TiO_2_ nanoparticles, resulting in some difficulty in the penetration of the electrolyte into the gaps. For TiO_2_-NF/MLG, TiO_2_ nanoflowers cause a big gap between the TiO_2_ nanoflowers, enabling relatively easy penetration of the electrolyte into the gaps. However, the top region of TiO_2_ nanoflowers has a long distance to exchange electrons with MLG, resulting in the difficult electron exchange of TiO_2_ with the electrode. With regard to TiO_2_-NR/MLG, intersected TiO_2_ nanorods form a 2D porous network parallel to MLG, which produces suitable nano-pores for electrolytes to reach TiO_2_ and a suitable distance for electron exchange between TiO_2_ and MLG, resulting in the highest capacity and rate capability.

The cycling performances of these samples at a current density of 0.1 A g^−1^ are shown in Figure 7b. In the initial five cycles, the capacities are delayed quickly, which may be due to the formation of SEI films and side reactions. Then, all samples maintain a rather stable specific capacity, and even a slight increase in capacity. Notably, TiO_2_ nanorods in TiO_2_-NR/MLG are composed of sub-sized TiO_2_ nanowires, leading to further penetration of the electrolyte in the interval of TiO_2_ nanorods and a gradual increase in the capacity. After 100 cycles, a specific capacity of 631.4 mAh g^−1^ can be maintained for TiO_2_-NR/MLG, which is higher than other composites. The TiO_2_-NR and MLG samples also provide stable specific capacities during the cycling tests, indicating the high stability of TiO_2_ and MLG for Li-ion anodes themselves. The capacity of TiO_2_-NR/MLG is much higher than those of MLG and TiO_2_-NR, indicating the best synergistic effect between MLG and TiO_2_ nanorods. Cycling performances of samples at a higher current density of 0.5 A g^−1^ are also displayed in Figure 7c. All TiO_2_/MLG samples exhibit high stability even at a high current density, indicating the high stability of the composites. Among them, the TiO_2_-NR/MLG sample exhibits the highest specific capacity of 456.5 mAh g^−1^ after 300 cycles with a capacity retention of 114.3%.

The kinetic process was tested to explain the advantage of the TiO_2_-NR/MLG composite. Figure 7d shows the EIS plots of TiO_2_-NR/MLG, TiO_2_-NR, and MLG electrodes after activation. All curves show a semicircle in the high-frequency range and an oblique line in the low-frequency range. The diameter of the semicircle reflects the charge-transfer resistance (Rct) and the oblique line represents the Warburg impedance (Zω) associated with Li-ion diffusion. An equivalent electric circuit shown in the inset of Figure 7d was used to fit these plots. TiO_2_-NR/MLG, TiO_2_-NR, and MLG display Rct values of 98.6, 646.7, and 1231 Ω, respectively. It shows that TiO_2_-NR/MLG has a lower charge-transfer resistance than both TiO_2_-NR and MLG due to the special combined nanostructure of MLG and TiO_2_, indicating the homogeneously distributed nanowires-assembled TiO_2_ nanorods on MLG not only accelerate the transfer of electron and Li-ion, but also improve the chemical activity of TiO_2_.

The comparison of the as-prepared TiO_2_-NR/MLG composite with previously reported TiO_2_-based composites on the cycling property is listed in Table 1. It can be seen that using the hydrothermal method to synthesize TiO_2_/graphene composites usually requires rather high temperatures of approximately 140~180 °C, a long reaction time of approximately 12~30 h, and post-annealing treatment at high temperatures of approximately 400~550 °C for 3~4 h. However, TiO_2_-NR/MLG can be synthesized by a simple chemical process at a low temperature of 90 °C for 7 h without post-annealing treatment in this work. It means that TiO_2_-NR/MLG can be synthesized with a higher efficiency under a lower energy cost, which is favorable for reducing the production cost of the composite. Moreover, the TiO_2_-NR/MLG electrode exhibits a higher reversible capacity and a higher rate performance when compared with these previously reported composites. It can be attributed to several special structural features of TiO_2_-NR/MLG, which contribute to higher electrochemical performances. Firstly, the carbon substrate of MLG is prepared via ultrasonic exfoliation and without further oxidation treatment, leading to less damage in the carbon bonds than graphene oxide. The higher conductivity of MLG can accelerate the movement of electrons and decrease the inner resistance of the electrode. Secondly, the 2D network composed of TiO_2_ nanorods on the MLG surface not only provides fast electron movement between TiO_2_ and MLG but also benefits the penetration of the electrolyte into the composite. Finally, the TiO_2_ nanorods are composed of nanowires, which can facilitate the electrolyte to penetrate the inner region of TiO_2_ nanorods. It further increases the contact surfaces of TiO_2_ with the electrolyte and the active sites of TiO_2_ in the electrode.

## 4. Conclusions

TiO_2_ was successfully synthesized on MLG surfaces by a soft chemical method, which did not need an activation treatment of MLG before synthesis. The morphologies of the synthesized TiO_2_ can be varied from anatase TiO_2_ nanoparticles to a mixture of anatase TiO_2_ nanoparticles and rutile TiO_2_ nanoflowers, rutile TiO_2_ nanoflowers, and rutile TiO_2_ nanorods by increasing the amount of HCl addition. Among them, the rutile TiO_2_ nanorods in TiO_2_-NR/MLG are composed of many smaller TiO_2_ nanowires, which contribute to the excellent electrochemical performance of the lithium-ion battery anode. A specific capacity of 631.4 mAh g^−1^ after 100 cycles at a current density of 100 mA g^−1^ was delivered. TiO_2_-NR/MLG has the advantages of cost-effective synthesis and high electrochemical performances.

## Figures and Tables

**Table 1:** Comparison of TiO_2_-NR/MLG with some reported TiO_2_-based composites on the cycling property.

TiO_2_ Based Materials	Synthesis Method	Cycle Capacity (mAh g^−1^)	Cycle Number (n)	Current Density (A g^−1^)	Publication Year	Reference
Mesoporous nanoplate TiO_2_/reduced graphene oxide	Hydrothermal treatment at 150 °C for 18 h	∼200	300	1.2C	2017	[18]
N-doped TiO_2_ nanotubes/TiN/graphene	Hydrothermal treatment at 160 °C for 48 h and heated at 500 °C for 3 h under Ar atmosphere	191.4	500	2	2019	[19]
R-TiO_2_/rGO	Hydrothermal treatment at 180 °C for 12 h, annealing at 500 °C for 4 h in a N_2_ atmosphere	267	100	1C	2019	[20]
		151	500	10C		
Reduced graphene oxide modified N-doped carbon foam-TiO_2_ (NCF@rGO-TiO_2_)	Soak of NCF@rGO in TBOT solution, hydrolysis of TBOT, calcination at 400 °C for 3 h in Ar atmosphere	214	150	1C	2019	[21]
		133.3	3000	10C		
TiO_2_ nanorods on reduced graphene oxide	Solvothermal process at 180 °C for 30 h and annealed treatment at 550 °C for 2 h under N_2_ atmosphere)	353.6	100	0.1	2019	[22]
Hybrid TiO_2_/Graphite/Nanodiamond	Microwave treatment method	540	100	0.5C	2021	[23]
		300	1000	5C		
TiO_2_ quantum dots confined in 3D carbon framework	Reverse microemulsion method combined with heat treatment	370.5	200	0.1	2021	[24]
Flower-like TiO_2_ hollow microspheres	Sol-gel method with a silicon dioxide (SiO_2_) template	121	1000	1	2022	[35]
TiO_2_ quantum dots on graphene nanoribbons	Hydrolysis strategy followed by heat-treatment	320.8	100	0.5	2022	[25]
Rutile TiO_2_ nanorods grown on carbon nanotubes	Microwave-assisted hydrothermal method	138.3 (1–3 V)	200	1C	2022	[26]
TiO_2_-NR/MLG	Chemical deposition at 90 °C	631.4	100	0.1		This work
		456.5	300	0.5		

## Data Availability

Data is contained within the article.
